# The contribution of brain sub-cortical loops in the expression and acquisition of action understanding abilities^☆^

**DOI:** 10.1016/j.neubiorev.2013.07.016

**Published:** 2013-12

**Authors:** Daniele Caligiore, Giovanni Pezzulo, R. Chris Miall, Gianluca Baldassarre

**Affiliations:** a Istituto di Scienze e Tecnologie della Cognizione, Consiglio Nazionale delle Ricerche (ISTC-CNR), Via San Martino della Battaglia 44, I-00185, Rome, Italy; b Istituto di Linguistica Computazionale “Antonio Zampolli”, Consiglio Nazionale delle Ricerche (ILC-CNR), Via Giuseppe Moruzzi, 1, I-56124, Pisa, Italy; c Behavioural Brain Sciences, School of Psychology, University of Birmingham, Edgbaston, Birmingham B15 2TT, UK

**Keywords:** Psychology, Neuroscience, Mirror neurons, Action understanding, Sub-cortical processes, Cerebellar cortical loops, Basal ganglia cortical loops, Forward models, Inverse models

## Abstract

Research on action understanding in cognitive neuroscience has led to the identification of a wide “action understanding network” mainly encompassing parietal and premotor cortical areas. Within this cortical network mirror neurons are critically involved implementing a neural mechanism according to which, during action understanding, observed actions are reflected in the motor patterns for the same actions of the observer. We suggest that focusing only on cortical areas and processes could be too restrictive to explain important facets of action understanding regarding, for example, the influence of the observer's *motor experience*, the *multiple levels* at which an observed action can be understood, and the *acquisition* of action understanding ability. In this respect, we propose that aside from the *cortical* action understanding network, *sub-cortical processes* pivoting on cerebellar and basal ganglia cortical loops could crucially support both the expression and the acquisition of action understanding abilities. Within the paper we will discuss how this extended view can overcome some limitations of the “pure” cortical perspective, supporting new theoretical predictions on the brain mechanisms underlying action understanding that could be tested by future empirical investigations.

## Highlights

• Focusing on cortical areas is too restrictive to explain action understanding ability.
• We propose that sub-cortical areas support action understanding ability.
• Cortical and sub-cortical processes allow acquisition of action understanding ability.

## 1 Introduction

In the last two decades, the study of action understanding in cognitive neuroscience has been revolutionized by the discovery of mirror neurons (Rizzolatti et al., 2001; Rizzolatti and Craighero, 2004; Lestou et al., 2008; Evangeliou et al., 2009; Bonini and Ferrari, 2011). Mirror neurons were first discovered in the monkey premotor area F5 (Di Pellegrino et al., 1992; Rizzolatti et al., 1996). Subsequently they were also found in the inferior parietal lobe (Fogassi et al., 2005; Gallese et al., 2002), in particular in area PFG (Rozzi et al., 2008; Bonini et al., 2010). The distinctive feature of these neurons is that they are activated when monkeys perform an action and also when they observe a similar action executed by another subject. Neuroimaging evidence suggests that mirror neurons might exist in homologous areas of the human brain (Buccino et al., 2001; Grèzes et al., 2003; Rizzolatti and Arbib, 1998; see also Mukamel et al., 2010 for a recent single cells recording in human patients with intractable epilepsy).

There is now a growing consensus that mirror neurons are part of a wider “action understanding network” in the monkey and human brain which, at minimum, encompasses the bilateral posterior superior temporal sulcus (STS) and adjacent middle and superior temporal gyri (MTG, STG, respectively), the inferior parietal lobule (IPL), inferior frontal gyrus (IFG), dorsal premotor cortex (PMd) and ventral premotor cortex (PMv) (Grafton and Tipper, 2012; Caspers et al., 2010; Cattaneo and Rizzolatti, 2009; Rizzolatti and Craighero, 2004). Within this cortical network, one hypothesis is that mirror neurons implement a “direct resonance” or “direct matching” mechanism according to which the observed action is “reflected” in the motor patterns for the same action of the observer (Buccino et al., 2004; Uithol et al., 2011). A related hypothesis, called “simulation theory” (Gallese and Goldman, 1998), is that embodied simulations support the encoding of perceived actions based on one's own motor repertoire (see also Prinz, 2006; Pezzulo, 2011; Pezzulo et al., 2013; Wilson and Knoblich, 2005; Wolpert et al., 2003 for related theories emphasizing prediction).

Three main issues feed the discussion about the limits of the mirror mechanism in explaining action understanding. First, how do the *motor abilities of the observer* and the *environmental constraints* contribute to action understanding? Both the cortical direct matching hypothesis and the simulation theory suggest that action comprehension crucially relies on the ability to produce the same action. However, these mechanisms alone might not be sufficient to support the understanding of actions that the perceiving agent cannot produce (Calvo-Merino et al., 2006; Press et al., 2011a). As an alternative to simulation theory, the “teleological theory” connected to it describes action understanding as an inferential process that operates over the target goal and the environmental constraints (contextual information) that might facilitate or limit the goal achievement. Although the result of this process may activate the motor system, the process in itself depends on non-motor mechanisms and then extends naturally to actions outside the motor repertoire of the perceiver agent (Csibra, 2003). In the same line, others have proposed that motor phenomena during action observation could be epiphenomenal rather than causal, and that detecting motor system activity during action observation does not license the conclusion that motor system is causally involved in action understanding (Mahon and Caramazza, 2008). They claim that it might be equally plausible that action understanding involves mainly perceptual processes and that, once action is understood, it activates the motor system which provides the information on how to (eventually) perform the action.

Second, the cortical mirror mechanism conceived as direct-matching and the simulation theory alone might not be sufficient to account for the *different levels at which an observed action can be understood* (Ramnani and Miall, 2004; Kilner, 2011). Action representations in the brain, indeed, are organized at multiple hierarchical levels and, as a consequence, there are multiple levels at which the observer could understand them (Hamilton and Grafton, 2007; Thill et al., 2013). As one moves up the hierarchy, the action is represented in more abstract terms (Kilner et al., 2007; Kilner, 2011; Pezzulo and Dindo, 2011). In particular, the kinematic aspects of a movement related to the trajectory and to the velocity and the motor aspects related to the muscle activity could be considered at the bottom of the action representations hierarchy, whereas the aspects related to the goal of the action (the purpose of the action, e.g., grasp an object) and to the intention of the action (the overall reason, e.g., grasp an object to eat) could be considered at the top of the hierarchy. A clear hypothesis on how the mirror neurons deal with action understanding at any level of complexity of action representation is still missing (Kilner, 2011). In the past some authors suggested a classification of mirror neurons based on different aspects of action representations analyzing the single neuron activation in primates. Gallese and colleagues (Gallese et al., 1996), for example, recorded the electrical activity from 532 neurons in the rostral part of inferior area 6 (area F5) of two macaque monkeys and used as a classification criterion the congruence between the executed and observed motor acts effective in triggering them, to split the mirror neurons into two main classes: strictly congruent and broadly congruent mirror neurons. Strictly congruent mirror neurons discharge when the observed and executed effective motor acts are identical both in terms of goal (e.g., grasping) and in terms of the way in which that goal is achieved (e.g., precision grip), whereas to be triggered broadly congruent mirror neurons require similarity but not identity between the observed and executed effective motor acts (Fabbri-Destro and Rizzolatti, 2008; Gallese et al., 1996). Some authors suggest that within the mirror circuit premotor and parietal areas could be respectively involved in the dissociable processing of abstract goals and movement representation at the kinematic level (Iacoboni et al., 2001; Iacoboni, 1999), whereas others suggest processing of action goals independent of motor trajectories in the parietal cortex (Fogassi et al., 2005; Hamilton and Grafton, 2006) and unexpected intentional actions in the STS (Saxe et al., 2004; Pelphrey et al., 2004). Molnar-Szakacs et al. (2006), for example, used fMRI to investigate the role of the fronto-parietal human mirror neuron system in representing hierarchical complexity during the observation of object-directed action sequences. They found that activity in mirror neuron areas varied according to the motoric complexity of the observed actions, but not according to the developmental sequence of action structures. Their results show how the fronto-parietal mirror neuron system provides a fairly accurate simulation process of observed actions, mimicking internally the level of motoric complexity. Finally, a further proposal suggests a two-pathway model to understand more abstract actions (those related to goals and intentions) that also involves ventral regions beside the traditional mirror circuit (Kilner, 2011; Press et al., 2011b). According to this view multiple possible actions are selected and processed during action observation, but one is represented more strongly than the others.

A third important issue regards the *acquisition* of the action understanding capacity. This point is crucial since a substantial part of the mirror circuit functioning might be related to the need to support the acquisition of action understanding ability and not only its expression. This claim is in line with the general idea that much of the structure and organization of the brain depends on the fact that behaviour has to be acquired, and not only expressed (Büchel et al., 1999; Xu et al., 2009; Bassett et al., 2010; Anderson, 2010; Caligiore et al., 2010).

**Fig. 1:**
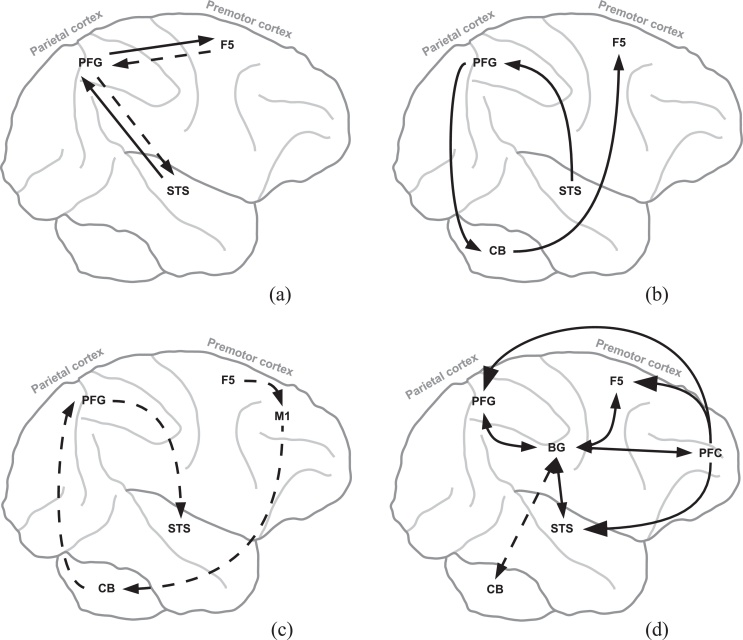
(a) Internal models within the mirror circuit. During action observation the circuit linking STS to F5 (STS-PFG-F5, solid arrows) may work as an inverse model, whereas during action execution the converse route from F5 to STS (F5-PFG-STS, dashed arrows) may act as a forward model (Miall, 2003). The inverse (b) and forward (c) models circuits within the mirror circuit and involving the cerebellum (CB). (d) Selection network within the mirror circuit involving basal ganglia (BG) and prefrontal cortex (PFC). The basal ganglia–cortical loops underlies actions competition within the mirror circuit (small-head arrows) whereas the PFC supplies the bias signal to select actions (large-head arrows). The basal ganglia also communicates with cerebellum through a bidirectional channel (dashed arrows).

The *associative sequence learning model (ASL*) proposes that we are not born with a mirror neuron system (Heyes, 2010). Rather, the mirror properties of mirror neurons emerge through *sensorimotor associative learning* where the natural correlation between observation of an action and its execution establishes excitatory links between sensory and motor representations of the same action. In this way, representations that were originally motor become mirror, that is activated when observing and executing the same action. According to the ASL hypothesis mirror neurons, as a by-product of associative learning, could play a part in action understanding but they do not develop for action understanding (Heyes, 2010; Press et al., 2011b). In contrast, some authors have recently proposed that the ASL hypothesis could support an explicit role of mirror neurons in action understanding if the hypothesis is considered within the *predictive coding* (PC) framework (Kilner et al., 2007; Urgesi et al., 2007). The PC proposal pivots on the *hierarchical* anatomical and functional organization of the cortical mirror circuit (STS, PFG, F5; Fig. 1a). According to the PC hypothesis, the activity within one level of the hierarchical organization of actions within the mirror circuit (Hamilton and Grafton, 2007) acts as a prior constraint on sub-ordinate levels. For example, contextual cues generate a prior expectation about the goal of the person we are observing. This prior expectation allows us to *predict* their motor commands, which in turn allow us to predict their kinematics on the basis of our own action system. The comparison of these predicted kinematics with the observed kinematics generates a *prediction error* that is used to update our representation of the person's motor commands. The prediction error is sent back to the higher level of action representation to update it. Ultimately the inferred goals are updated by minimizing the prediction error between the predicted and inferred motor commands. By *minimizing the prediction error at all the action representation levels* of the mirror circuit, the most likely cause of the action will be inferred at all levels (intentions, goals, motor commands, kinematics). This approach provides an hypothesis on how the cause of an action can be inferred from its observation (see also Friston et al., 2011). Both ASL and PC accounts are framed considering cortical processes (Press et al., 2011b) that alone might not fully support important functions required by those proposals (e.g., computation of a prediction error and learning based on its minimization).

In this article we propose that the three open issues we have discussed above could be better addressed considering the crucial role of *sub-cortical processes underlying action understanding*. Mirror mechanisms responsible for action understanding are, indeed, mainly studied at cortical levels almost ignoring how key sub-cortical areas, which strongly work in concert with several cortical regions including the regions forming the mirror circuit, could contribute to the functioning and to the formation of the mirror processes for action understanding. Here we will analyze the sub-cortical processes behind action understanding, pivoting on recent anatomical and functional evidence about the involvement of the *cerebellum* and the *basal ganglia* in motor and non-motor functions (Strick et al., 2009; Ito, 2008; Houk, 2011). The cerebellum and basal ganglia receive input from and send output to the cerebral cortex through multisynaptic loops that have been assumed to be anatomically segregated and to perform distinct functional operations (Alexander et al., 1986; Middleton and Strick, 1994, 2000; Houk et al., 1996).

After briefly recalling the “traditional” cortical viewpoint of the mirror circuit, which is the starting point of our analysis, we discuss how sub-cortical processes might influence the functioning of the mirror circuit (Section 2). Then we illustrate how these processes could contribute to the formation of the mirror circuit for action understanding (Section 3). Finally, in Section 4 we draw conclusions, highlighting the main points of our analysis and proposing some predictions for future empirical investigations.

## 2 Sub-cortical processes affecting mirror circuit functioning for action understanding

An elegant and effective explanation of how the cortical mirror circuit could work has been given by Iacoboni et al. (2001) and Miall (2003). They suggest that during action observation the circuit formed by the superior temporal sulcus (STS), the area (PF) of the inferior parietal lobe (taking into account the recent data provided by Rozzi et al. (2008) and Bonini et al. (2010) here we consider PFG rather that PF), and the ventral premotor cortex (F5) (*STS-PFG-F5*) works as an *inverse model*. This circuit transforms the visual representation of observed action encoded by the neurons of STS into a motor plan encoded by mirror neurons of F5 (Fig. 1a, solid arrows). Conversely, during action execution the reverse connections from F5 mirror neurons to PFG and in turn to STS work as a *forward model* that converts the motor plan back into a sensory outcome of action (i.e., a predicted visual representation encoded by neurons of STS, Fig. 1a, dashed arrows).

The cortical areas forming the mirror circuit do not work in isolation from sub-cortical structures. Rather, these areas form partially segregated *loops* with corresponding areas of the *cerebellum* (Middleton and Strick, 2000) and *basal ganglia* (Alexander et al., 1986). In this section we discuss how these loops might critically support the functioning of mirror neurons for action understanding. In particular, we focus on the two issues raised in the introduction about the role of the observer's motor experience and environmental constraints, and about the multiple levels at which an observed action can be understood.

### 2.1 The role of the cerebellum within the cortical action understanding network

As first highlighted by Miall in 2003 the schema shown on Fig. 1a has an important unresolved issue. It is based on internal model processes (both inverse and forward) without considering the key sub-cortical system that plays a critical role in building and functioning of internal models: the cerebellum (Miall, 2003; Wolpert et al., 1998). To deal with this issue, Miall (2003) proposed an alternative pathway within the mirror circuit which involves the cerebellum (Fig. 1b,c).

Thanks to the strong connections between parietal areas and the cerebellum (Schmahmann and Pandya, 1989; Stein and Glickstein, 1992; Middleton and Strick, 2000) which is in turn connected to the ventral premotor area (Strick et al., 2009), the major route for visuo-motor information to reach F5 might include the *STS-PFG-CB-F5* circuit. In this case the *cerebellum* might work as an *inverse model* (Fig. 1b). Moreover, the cerebellum could use the efferent copy of motor signals sent by the primary motor cortex (M1) neurons to work as a *forward model*, which entails a visuo-motor update to the motor representation within PFG (circuit *F5-M1-CB-PFG-STS* on Fig. 1c). In this way the mirror neurons in F5 may code a motoric representation of visuo-motor actions, both during action execution and observation, driven by the cerebellar internal models (Fig. 1b,c). Miall's proposal is well situated within the growing literature suggesting that the cerebellum could be crucially involved in the brain circuits underlying action understanding processes (Sokolov et al., 2010, 2012; Hamilton et al., 2006, 2007; Fuentes and Bastian, 2007). In this line, Cattaneo et al. (2012) have recently suggested that the role of the cerebellum as sequencer of motor acts (Molinari et al., 2008; Strick et al., 2009) could be exploited also in the action observation domain. They have shown that patients with lesions of the cerebellum perform worse than healthy subjects in a task that requires assembling pictures of individual observed motor acts into a meaningful sequence.

The involvement of the circuits including the cerebellum (Fig. 1b,c) might support action understanding even if the observer *lacks a motor routine* of the observed action. There are at least two ways to understand actions outside one's own motor repertoire that are consistent with the idea of a reuse of one's own motor processes and that could involve the cerebellum. The first is the use of forward models only (rather than also inverse models) to process the to-be-observed actions, or the use of predictive internal models that support perceptual exploration strategies associated to actions (Flanagan and Johansson, 2003). This implies becoming a “visual” (or more generally perceptual) expert without necessarily being able to reproduce the entire action. In support of this view, it has been suggested that forward models can be learned before inverse models (Flanagan et al., 2003).

The second possibility, discussed by Schubotz (2008), consists in generalizing the prediction abilities of the motor system beyond their initial domain of acquisition, that is, beyond the prediction of action effects. Neuroimaging studies show that premotor cortex is involved in the prediction of perceptual events in a somatotopic way. For example, premotor areas involved in hand movements are active during a task requiring the prediction of the size of circles; premotor areas involved in articulation are active during a task requiring the prediction of the pitch of a tone (Schubotz and von Cramon, 2002; Wolfensteller et al., 2007). It is therefore possible that premotor mechanisms are reused outside action prediction tasks as they successfully incorporate relevant statistical regularities; for example, the statistics used to predict articulation might also be used (to some extent) to predict nonverbal auditory stimuli. In a similar way, cerebellar mechanisms that incorporate useful action regularities could be reused outside their initial domain of acquisition.

**Fig. 2:**
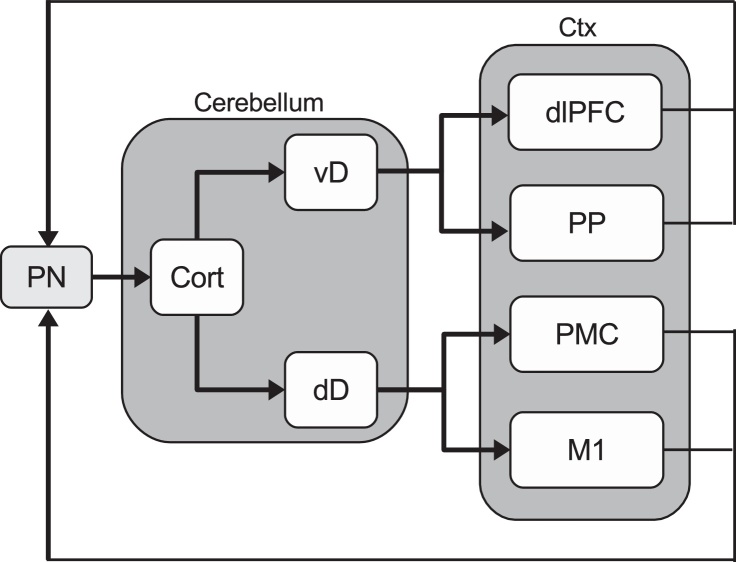
Sketch of the cerebellar cortical loops. The figure does not aim to supply a detailed overview of all the cerebellar components; rather, it focuses on the main interactions between the dentate output nuclei of the cerebellum and the target regions of the fronto-parietal cortex. PN: pontine nuclei; Cort: cerebellar cortex; vD: ventral dentate nucleus of the cerebellum; dD: dorsal dentate nucleus of the cerebellum; dlPFC: dorsal lateral areas of the PFC; PP: posterior parietal areas; PMC: premotor cortex; M1: primary motor areas; Ctx: cortex.

The cerebellum might also contribute to the issue related to the multiple levels at which an observed action can be understood. Many works, indeed, suggest that the cerebellum is involved in the communication between cortical areas such as the occipital, temporal and prefrontal ones, and some areas of the mirror circuit (Grèzes and Decety, 2001; Calvo-Merino et al., 2006; Middleton and Strick, 1996; Strick et al., 2009). In this respect, some relevant information is given by some aspects of cerebellar anatomy (Fig. 2). The cerebellum shares a set of discrete parallel loops with various parts of the fronto-parietal cerebral network (Dum and Strick, 2003; Middleton and Strick, 2000; Cisek and Kalaska, 2001; Strick et al., 2009). In particular, the output channels in the ventral dentate nucleus of the cerebellum project to the posterior parietal cortex and to the dorsal areas of the prefrontal cortex (PFC; areas 9 and 46), the latter involved in working memory and planning and a major site of termination of the “dorsal stream” of visual processing (Dum and Strick, 2003; Middleton and Strick, 2000). These cortical areas targeted by the dentate output also project back to the cerebellum via efferents to pontine nuclei (Fig. 2; Dum and Strick, 2003; Schmahmann, 1998; Middleton and Strick, 1996, 2000).

The cerebellar-dorsoprefrontal closed-loop circuits (Fig. 2) could represent a functional unit of cerebrocerebellar circuitry that could be important in forming and detecting high-level goals and intentions (see Introduction). This function is supported by the capacity of these areas to process information from various multimodal associative areas that allow them to represent and process the environmental context (Uithol et al., 2011; Kilner, 2011; Kim et al., 1994; Strick et al., 2009). In this respect, the PFC, *working in synergy with the cerebellum* (Middleton and Strick, 2000; Ito, 2008), might possess the capacity of anticipating future events at fast temporal scales based on forward models (Wolpert et al., 1995). Thus, if we consider goals and intentions as desired anticipated states (Matsumoto and Tanaka, 2004; Thill et al., 2013), the capacity of PFC to project into the future is an important prerequisite for the formation and detection of goals, as well as for biasing the activation of actions related to those goals within the mirror circuit.

The cerebellar-parietal loops supporting the coordination of action execution (Ramnani et al., 2001), might also play a role during action observation. In this case the signal coming from the ventral dentate output of the cerebellum might support the coordination between the dorsal areas of the PFC and the posterior parietal areas to represent high-level goals taking into account the temporal relationship between task-relevant events (D’Angelo and Casali, 2013). This view is in line with the “timing hypothesis” postulating that the cerebellum is critical for representing the temporal relationship between task-relevant events (Bower, 1997; Verschure and Mintz, 2001; Jacobson et al., 2008; D’Angelo and De Zeeuw, 2009; Yamazaki and Tanaka, 2009; Yamazaki and Nagao, 2012) as it works as a general timing co-processor whose effect depends on the targeted centres (see D’Angelo and Casali, 2013 for an excellent recent overview on this topic).

Other cerebellar loops pass through the primary motor cortex and premotor areas. In more detail, the output channels in the dorsal dentate nucleus of the cerebellum project to primary motor and premotor areas which, in turn, project back to the cerebellum via efferents to pontine nuclei (Fig. 2; Dum and Strick, 2003; Middleton and Strick, 2000). The loops with primary motor cortex are presumably involved in adjusting motor command to compensate for movement dynamics, whereas the further loops passing through premotor cortex, where mirror neurons are located, may be involved in predicting the immediate consequences of specific intended movements (Cisek and Kalaska, 2001; Haslinger et al., 2002). The observation of mirror neurons in ventral premotor areas (Fadiga et al., 2000) is consistent with the functions ascribed to cerebellar-premotor closed loop circuits (Fig. 2) related to the prediction of the immediate consequences of specific intended movements.

### 2.2 The role of the basal ganglia within the cortical action understanding network

Beside the cerebellum, action selection mechanisms pivoting on the basal ganglia cortical loops might support other complementary neural processes underlying the understanding of high-level actions. To clarify this idea, a good starting point is the “affordance competition” hypothesis put forward by Cisek (Cisek, 2007; Cisek and Kalaska, 2010). According to this proposal, the parietal-premotor circuit specifies multiple action plans based on available affordances, which compete for selection until one is chosen to be overtly executed (Caligiore et al., 2010, 2013). Within this parallel specification and selection scheme, the dorsal stream processes inputs from sensory stimuli and contributes to the specification of action and to preparatory motor processing. The ventral stream recognizes object identities and elicits associated internal motivational and affective processes based on amygdala, PFC, and ventral portions of the basal ganglia (see Yin and Knowlton, 2006 and Mirolli et al., 2010). In turn, this permits behavioural biasing and the evaluation of the concurrent motor plans.

Selection mechanisms such as those described by the affordance competition hypothesis might participate in managing high-level actions understanding. In more detail, during action observation multiple possible actions are processed but only few are represented more strongly than the others and are selected (Kilner, 2011). The selection among neural representations of many different actions within F5 and PFG (Fogassi et al., 2005; Hamilton and Grafton, 2007; Chersi et al., 2011; Dindo et al., 2011) could be driven by a bias signal generated by PFC, whose neurons encode goals and intentions (Thill et al., 2013). In this way goals and intentions might be actually translated into *specific* actions via a strong bias exerted onto representations encoded by mirror neurons within F5 and PFG. The selection process leads to a competition among different actions within F5 and PFG, based on reciprocal inhibition of competing neural populations, and this competition might take place within whole circuits formed by basal ganglia-cortical loops (Redgrave et al., 1999a, 1999b; Kandel et al., 2000; Cisek and Kalaska, 2005). Fig. 1d shows BG-F5 and BG-PFG circuits underlying the integration of information from various sources (Weber and Yin, 1984; Glickstein, 2003) and supporting PFC signals to drive action selection mechanisms (Cisek and Kalaska, 2010; Pezzulo and Castelfranchi, 2009).

**Fig. 3:**
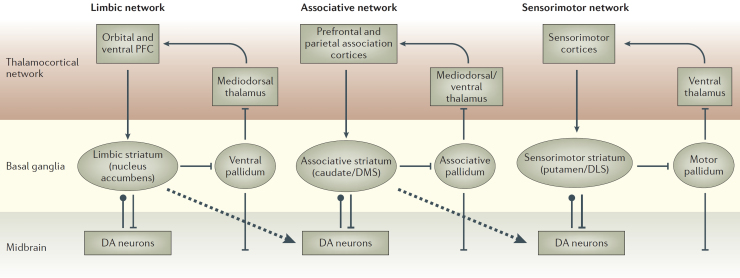
Sketch of the three main cortico-striatal regions and their interconnections. Standard arrows indicate excitatory glutamate connections. Flat arrowheads indicate inhibitory GABA connections. Dot arrowheads indicate dopaminergic connections whereas dashed arrows indicate cross-loop connections. Reprinted with permission from Mcmillan Publishers Ltd: Nature Reviews Neuroscience, Yin and Knowlton (2006), copyright 2006.

Analyzing the hierarchical anatomical and functional organization of the basal ganglia-cortical loops could be crucial to explain how the action selection mechanisms operate. In more detail, three distinct functional domains can be distinguished within the basal ganglia corresponding to the dorsolateral striatum (DLS), dorsomedial striatum (DMS), and ventral striatum (VS), the latter also called the nucleus accumbens (Fig. 3) (Yin and Knowlton, 2006; Baldassarre et al., in press). Such domains are identifiable in rats and mice and are homologues to respectively the putamen, caudatum, and nucleus accumbens in primate striatum. These different loops run in parallel and each loop starts from a cortical area, goes through a subregion of the basal ganglia, and goes back to the cortical area of origin via the thalamus, forming distinct multiple re-entrant loops interacting with distinct portions of cortex, including the areas of the mirror circuit (Alexander et al., 1986; Humphries and Prescott, 2010; Middleton and Strick, 2000; Romanelli et al., 2005). Within each one of these streams there is evidence of the existence of relatively segregated channels capable of selecting particular cortical restricted targets (Chevalier and Deniau, 1990; Gurney et al., 2001; Mink, 1996). In particular, based on these channels, each loop is thought to be involved in the selection of the content of the targeted cortical areas, such as a perceptual representation, an action, or a goal (Alexander and Crutcher, 1990; Redgrave et al., 1999b).

The distinct loops typically play different functional roles depending on the type of information processed within the targeted cortex, and on this basis are also respectively called the sensorimotor, associative and limbic loops (Yin and Knowlton, 2006; Fig. 3). The cortical areas that reciprocate connections with the nucleus accumbens are the ventro-medial, orbitofrontal, and dorsolateral portions of the PFC, important for the processing of biologically salient states and outcomes (Humphries and Prescott, 2010; Voorn et al., 2004; Zahm, 2000). In general, the limbic loop is involved in the selection of final goals (e.g., the achievement of a certain food), and means-to-end goals (e.g., opening a door to access a lever activating a food dispenser), based on motivations. The limbic loop is also important for reward and motivation based on dopamine regulation (Berridge and Robinson, 1998; Corbit and Balleine, 2011).

The cortical areas that reciprocate connections with the caudatum are the temporal cortex (Middleton and Strick, 1996), the parietal cortex, the frontal eye-fields, and the dorsal regions of the PFC (Alexander et al., 1986; Voorn et al., 2004). The associative loop is involved in the formation of high-level visual representations (typically processed in temporal areas; Middleton and Strick, 1996), in attention, spatial orientation, and affordance selection (involving frontal eye field and parietal areas, Schrimsher et al., 2002; Volkow et al., 2007), and in working memory tasks (involving various areas of the PFC; Levy et al., 1997; Lewis et al., 2004).

Finally, the putamen is in a sensorimotor loop with motor cortex (MC), premotor cortex (PMC), and supplementary motor cortex (SMC) (Romanelli et al., 2005; Tang et al., 2007). There is clear evidence that the sensorimotor loop is involved in the selection of final sensorimotor mappings based on the current context (Alexander et al., 1986; Romanelli et al., 2005; Yin and Knowlton, 2006).

The three striato-cortical loops form a functional hierarchy (Yin and Knowlton, 2006; Baldassarre et al., in press). Each loop collects a rich set of information from various cortical areas and on this basis selects the contents processed in a specific target cortical area within this hierarchy. The decisions about motor actions, supported by the sensorimotor loop, are at a lower level with respect to the decisions about the part of the current context the animal should attend and process, which relies on the associative loop. In their turn, the latter decisions are at a lower level with respect to decisions about the motivationally salient outcomes (the high level goals of the animal) processed by the limbic loop. The selection processes performed by basal ganglia have an increasing importance (e.g., in terms of neural resources involved and in turn in terms of neural activation) going towards the higher levels of the hierarchy. Thus, at the highest level of the hierarchy the VS, supplied with rich information on value of stimuli by various sub-cortical areas (e.g., amigdala, hippocampus, hypothalamus), contributes to select biologically relevant goals encoded in the orbitofrontal, dorsolateral and ventromedial prefrontal cortices. At a lower level, the DMS contributes to select more abstract goals encoded in the dorsolateral PFC. At the lowest level, the DLS selects motor plans, encoded in PMC, and action implementation processes, encoded in the primary motor cortex. Importantly, this functional hierarchy is supported by a top-down parallel anatomical hierarchy involving the so called striato-nigro-striatal *spirals* that start from the accumbens, continue through the caudatum, and terminate in the putamen, and that are important to transfer motivational information related to goals and homeostatic drives processed in the limbic loop downstream to the associative and sensorimotor loops (Haber et al., 2000; see also Fig. 3).

The output of the basal ganglia could also influence higher order levels of visual processing. Basal ganglia could support STS cells to respond selectively to the observed body movements. STS is indeed a source of input to the basal ganglia, but also their target (Fig. 1d; Maioli et al., 1983; Middleton and Strick, 1996). The same connection pattern repeat for the temporal lobes. As a consequence of the influence of the basal ganglia, the areas of temporal lobes might further elaborate the visual signals to better identify the object identity (cf. Cisek, 2007; Caligiore et al., 2010; Thill et al., 2013). Within the mirror system, the visual input concerning biological motion coming from STS is mainly fed to PFG, where mirror neurons are mostly present, and to AIP (Rozzi et al., 2006; Nelissen et al., 2011). During action observation the neurons of area STS respond selectively to a range of body movements (Oram and Perrett, 1994; Grossman et al., 2000; Hoffman and Haxby, 2000; Keysers and Perrett, 2004; Oztop and Arbib, 2002) and this function might be supported by the selection mechanisms conveyed by the basal ganglia-STS circuits, as suggested by their anatomy and functionalities, based on the pattern of repeated striato-thalamo-cortical loops described above.

Including the selection processes of basal ganglia within the mirror circuit might also help to interpret recent fMRI data which show that mirror neurons are modulated by the motivational state (Cheng et al., 2007). These data show how human participants who observe grasping actions directed at food exhibit a higher activation of premotor and parietal mirror areas when they are hungry than when they are satiated, indicating that the mirror system is sensitive to the needs and drives of the participants. Moreover, the same scans show a higher activation of orbitofrontal cortex as well as amygdala and hippocampus. This indicates that the orbitofrontal cortex, the PFC area connected with sub-cortical limbic systems such as amygdala and hippocampus known to interface the brain with the visceral body, might be the origin of the observed modulation of mirror neuron activity. The selection among alternative appetitive outcomes on the basis of current needs and motivational states, which pivots on the limbic basal ganglia-cortical loop, might be the mechanism underlying the observed modulation of mirror neurons activity.

### 2.3 Basal ganglia-cerebellar-cortical neural circuits underlying action understanding

**Fig. 4:**
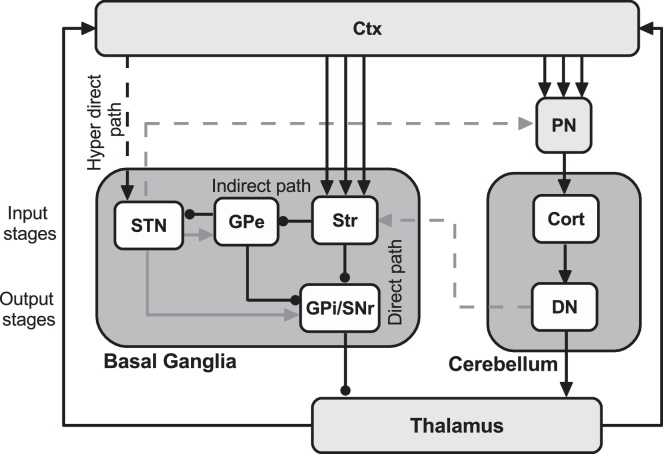
Schema of the neural circuits proposed to be mainly involved in action understanding processes. The dashed black arrow indicate the hyper direct pathway linking the subthalamic nucleus (STN) with the cortex (Ctx). The dashed gray arrows indicate the circuits interconnecting basal ganglia and cerebellum: an output stage of cerebellar processing, the dentate nucleus (DN), has a disynaptic connection with an input stage of basal ganglia processing, the striatum (Str) (Hoshi et al., 2005); there is also a reciprocal connection from the STN to the input stage of cerebellar processing, via pontine nuclei (PN), the cerebellar cortex (Cort) (Bostan et al., 2010, 2013). These interconnections enable two-way communication between the basal ganglia and cerebellum. Each of these subcortical modules receives signals from several areas of cerebral cortex through separate parallel channels (parallel solid black arrows) and sends signals (through Thalamus) to the cerebral cortex. The gray arrows and the black lines ending with a filled circle represent excitatory glutamatergic and inhibitory GABAergic projections, respectively. GPi, internal segment of the globus pallidus; GPe, external segment of the globus pallidus; SNr, substantia nigra pars reticulata. Some aspects of the schema arrangement are based on the proposals and results presented in Nambu et al. (2002), Hoshi et al. (2005), Bostan et al. (2010, 2013), and Baldassarre et al. (in press).

Pivoting on recent data on the anatomical and functional organization of basal ganglia and cerebellum and on their interactions with mirror cortical areas, we propose a novel schema on the cortical-subcortical neural circuits that might be involved in action understanding. In more detail, our perspective is mainly based on the neural circuits comprising the subthalamic nucleus (STN) of the basal ganglia (Alegre et al., 2010; Marceglia et al., 2009), the reciprocal disynaptic pathways between the basal ganglia and the cerebellum (Hoshi et al., 2005; Bostan et al., 2010, 2013), and the sub-cortical loops these areas form with the mirror cortical areas (see Section 2). Fig. 4 summarizes the connectivity between these areas.

The STN is part of the closed ancillary loop in the basal ganglia circuitry including the external globus pallidus (GPe). The striatum (Str) is the input part of the basal ganglia receiving direct excitatory cortical inputs. Str projects to the output nuclei of the basal ganglia formed by the internal globus pallidus (GPi) and the substantia nigra pars reticulata (SNr) through two major projection pathways (Alexander and Crutcher, 1990; Albin et al., 1989). The first pathway (”direct pathway”) arises from GABAergic striatal neurons, and projects monosynaptically to the GPi/SNr. The second pathway (”indirect pathway”) arises from GABAergic striatal neurons, and projects polysynaptically to the GPi/SNr by way of a sequence of connections involving the GPe and STN. In this way, STN assumes a crucial position as a relay nucleus of this second pathway. Importantly, STN could be regarded as another input station of the basal ganglia besides the Str as it receives direct cortical projections, especially from the frontal lobe (Nambu, 2004) and sends outputs to the GPi/SNr (“hyper-direct” pathway; Fig. 4).

Cortical activity triggered in the mirror circuit by movement observation may therefore easily propagate to the basal ganglia and, in particular, to the STN through these pathways. Recent evidence supports this view (Alegre et al., 2010; Marceglia et al., 2009; Castiello et al., 2009). The input from mirror cortical activity, reflected in the changes observed in the STN local field potentials (Alegre et al., 2010), might serve to modulate basal ganglia output according to the predicted or more likely outcome in terms of action. The basal ganglia output might in turn modulate the response of the mirror cortical circuits during action observation (Marceglia et al., 2009). This modulation pivots on the selection mechanisms supported by the activation of STN and might be necessary for the deployment of additional activity which could be necessary to influence cortical functions related to the representations of observed actions (Alegre et al., 2010; Castiello et al., 2009).

The management of the timing of the communication between the STN and the premotor areas is a critical point highlighted in the literature (Marceglia et al., 2009). In this respect, we propose that the cerebellum might assist this communication by providing accurate timing of series of signals coming from the cerebral cortex and the basal ganglia. The cerebellar-cortical loops discussed above, indeed, might be involved in the time integration of the current, memorized, and predicted sensory information in order to allow effective action selection (Cisek and Kalaska, 2001; D’Angelo and Casali, 2013). In this perspective it might be also important to consider recent empirical literature providing data about the anatomical substrate for a bidirectional communication between basal ganglia and cerebellum (Fig. 4 and also Fig. 1d, dashed arrows; Hoshi et al., 2005; Bostan et al., 2010, 2013). The STN of the basal ganglia has an important disynaptic projection to the cerebellar cortex. This pathway provides a means for signals from the basal ganglia to influence cerebellar function (Bostan et al., 2010). Moreover, the dentate nucleus of the cerebellum has a disynaptic projection to the main input stage of basal ganglia, the striatum (Hoshi et al., 2005). These recently discovered bidirectional communication channels between the cerebellum and the basal ganglia might also support the timing functions of the cerebellum. In general, this view is in line with the timing hypothesis discussed above in Section 2, according to which the cerebellum could be seen as a general timing co-processor whose effect depends on the targeted centres (D’Angelo and Casali, 2013).

Remarkably, the schema showed on Fig. 4 could be used to explain recent data suggesting that the influence of the mirror system on the facilitation of voluntary movements is altered in Parkinson's disease (PD) (Tremblay et al., 2008; Castiello et al., 2009). In general, visual stimuli facilitate the execution of voluntary movements in PD and this facilitation occurs both with “biological” movement observation and with action-related objects observation (Poliakoff et al., 2007). However, in a task where PD patients had to react to the observation of an action which they were subsequently requested to perform, they showed facilitation effects only when the subject they observed was a Parkinsonian patient, that is when the observed action matched the action they could perform. When the patients observed a healthy subject (non matching condition), the facilitation effects disappeared (Castiello et al., 2009). In such circumstances, damage to the structures shown in Fig. 4 might prevent the deployment of additional activity which might be necessary to influence cortical functions related to the representations of observed actions.

## 3 The role of subcortical processes in the development of the mirror circuit for action understanding

How does action understanding *develop* within the mirror circuit? This is a cardinal question which needs to be addressed. The whole organization and functioning of the mirror circuit might, indeed, be related to the need to support the acquisition of action understanding behaviour and not only its expression (Caligiore et al., 2010).

The combination of the *associative sequence learning model* (*ASL*) and *predictive coding* (PC) account offers a possible answer to this question. Once mirror neurons are acquired by an ASL process, the PC process uses information supplied by the mirror neurons about which goals are most likely given a certain intention, and which kinematics are most likely given a certain goal, to test hypotheses about the observed actors’ actions. In this way, the description of a behaviour at one level within the brain hierarchy acts as a prior constraint on subordinate levels. The mismatch error between expectations and observations at each level is at the basis of the learning of the forward models incorporated in the hierarchy.

Both *ASL* and *PC* models mainly refer to *cortical mechanisms* (Press et al., 2011b) that, alone, might not be sufficient to support the development of mirror neurons for action understanding (e.g., the computation of a prediction error and learning based on its minimization). In this section we will see that including sub-cortical processes pivoting on basal ganglia- and cerebellar-cortical loops crucially refines and develops the ASL and PC theories under a number of aspects, briefly discussed below.

*Beyond associative learning to acquire action understanding capacity.* In agreement with the ASL perspective a simple account of the development of the mirror mechanism suggests that plasticity of connections between the three core cortical areas of the mirror circuit, STS, PFG, and F5 is regulated by a Hebbian associative mechanism (Keysers and Perrett, 2004; Chersi et al., 2011). However, action understanding is a complex process including both motor and cognitive aspects of behaviour; hence it is unlikely that it develops based only on simple Hebbian associative learning (Press et al., 2011b). The acquisition of the action understanding capacity by mirror neurons (e.g., to minimize prediction errors according to the PC hypothesis) might require that the associative Hebbian learning process is paralleled by more powerful learning processes, in particular by supervised learning.

In this respect, it might be important to consider the proposal according to which the brain organization is based on three major divisions distinguished by the learning algorithms they implement (Doya, 2000; see also Shadmehr and Krakauer, 2008; Izawa and Shadmehr, 2011): (a) the cerebral cortex, learning based on unsupervised associative mechanisms (mainly of Hebbian type); (b) the cerebellum, learning based on supervised learning; and (c) the basal ganglia, learning based on trial-and-error processes (reinforcement learning). As cortical areas work in concert with cerebellar and basal ganglia structures, we suggest that the development of action understanding is based not only on the unsupervised Hebbian cortical learning processes but also on cerebellar supervised learning and basal ganglia trial-and-error learning.

*Basal ganglia-cortical loops support action selection needed by predictive coding.* The PC account suggests that one level within the hierarchical organization of actions (Hamilton and Grafton, 2007) acts as a prior constraint on sub-ordinate levels. The contextual cues used to generate a prior expectation about the intention of an observed actor bias the *selection* of an action at lower levels within the hierarchical organization of actions. The biasing contextual cues might be conveyed by PFC, whereas the selection of an action within level might be based on a dynamical competition among different actions encoded within F5 and PFG. The competition depends on reciprocal inhibition of competing clusters, representing different actions, which might be supported by whole systems formed by the basal ganglia-F5 and basal ganglia-PFG loops (Fig. 1d; Redgrave et al., 1999a; Cisek and Kalaska, 2005).

*Cerebellar-cortical loops support “Predictive Coding programs”, computing and minimizing prediction errors.* According to the PC framework the activity within one level of the hierarchical organization of action within the mirror circuit predicts representations in the level below, via the forward model circuit involving F5-PFG-STS (Fig. 1a, dashed arrows and cf. Press et al., 2011b). For example, a prior expectation about the goal of the observed person allows an observer to predict the motor commands that the person will perform, which in turn allows the agent to predict the related kinematics. These predictions are compared with the representations at the subordinate action level to produce a prediction error. This prediction error is then sent back up to the higher level, via the inverse model circuit STS-PF-F5 (Fig. 1a, solid arrows), to update action representation at this higher level. For example, the comparison of the predicted kinematics with the observed kinematics generates a prediction error that is used to update the representation of the person's motor commands. By minimizing the prediction error at all levels of action representation within the mirror system, the most likely cause of the action will be inferred at all levels. Thus, ultimately the inferred goals are updated by minimizing the prediction error between the predicted and inferred motor commands.

The associative Hebbian mechanisms within the cortical areas STS, PFG, F5 might not be suitable to generate and minimize prediction errors as required by the PC hypothesis. Rather, both the production and the minimization of the prediction error might involve cerebellar cortical loops and *supervised learning processes* working in synergy with associative cortical processes (Doya, 2000; Houk, 2011; Shadmehr and Krakauer, 2008; Izawa and Shadmehr, 2011). In this line, we propose that the *cerebellar forward/inverse circuits* (Fig. 1b,c) might be firstly involved in implementing the PC processes leading to the *acquisition* of the action understanding capacity by the mirror neurons. The supervised learning processes pivoting on cerebellum, indeed, might contribute to the minimization of the prediction errors at all level of action representation.

This account suggests that the supervised learning pivoting on cerebellar circuits might allow both cerebellar (Fig. 1b,c) and cortical forward and inverse models (Fig. 1a) to acquire the *PC programs* (i.e., the processes computing and minimizing the prediction errors given a certain context). During practice, as the PC programs are acquired, they are transferred to premotor and parietal areas for more efficient and faster execution (Hua and Houk, 1997; Koziol et al., in press). This transfer process might work as an automatization process, according to which the cerebellum exports some routine cognitive functions to the cerebral cortex to improve the efficiency of behaviour and thought (Hua and Houk, 1997; Koziol et al., in press; and see Raichle et al., 1994, for PET studies showing that metabolic activity associated with a cognitive task moves from the cerebellum to cortex with practice). Thus, once the main PC programs are acquired they could be executed by cortical premotor and parietal areas whereas the cerebellar circuits might remain important as a “reservoir” of PC programs. The enormous storage capacity of the cerebellum (Tyrrell and Willshaw, 1992; Hua and Houk, 1997) might support this function of the cerebellar circuits. According to this view not only do anticipatory control loops between cerebellum and cortical areas support actions processes, but they also permanently structure and sculpt cortical representations (Koziol et al., in press). Once the learning process related to a specific forward/inverse model in cortical circuits is determinated (Fig. 1a), cortex is able to work without further involvement of the cerebellar-cortical forward/inverse circuits (Fig. 1b,c). Elimination of cerebellar involvement is advantageous as it shortens the control pathway for responses that are used frequently, thus forming what has been referred to as “sensorimotor habits” (Hua and Houk, 1997). This shift in function from cerebellum to cortex could be a potential source of confounds in the empirical study on the role of cerebellum in the mirror circuit functioning.

*Cerebellar cortical loops support the detection of others’ actions.* Detecting that an observed action is performed by another individual is a first step that the observer must take during the acquisition of action understanding. The cerebellum might give an important contribution to this process by updating the perceptual predictions about the sensory consequences of one's own action. This function is accomplished by comparing internal predictions about the sensory consequences of our own actions with the actual afference, thereby isolating the afferent component that is externally produced (Wolpert et al., 1998; Haarmeier et al., 2001). The cerebellum might mediate the updating of predictions about the sensory consequences of actions, ensuring both precise action performance and truthful perceptual interpretation of external events, including actions (Wolpert et al., 2003; Synofzik et al., 2008). When sensory input from the environment is not due to one's own action but to an action of another individual, the cerebellum should not update its perceptual predictions about the sensory consequences of actions. In more detail, the afference is compared with an internal prediction about the sensory consequences of one's own action generated by the cerebellar forward model circuit, F5-M1-CB-PFG-STS (Fig. 1c). The internal prediction is based on signals related to movements, including efference copy of the motor command and sensory information of the state of the body (Wolpert et al., 2003). In case of a match between the internal predictions about the sensory consequences of one's own actions and afference, the afference can be interpreted as a result of self-action (reafference). In the case of a mismatch between the internal predictions about the sensory consequences of one's own actions and afference, the difference corresponds to an external sensory event, and any observed action must have been performed by another person (Frith et al., 2000; Synofzik et al., 2008). Thus, the mismatch between the internal predictions about the sensory consequences of one's own actions and afference might supply a “go signal” to inform the STS-PFG-CB-F5 circuit that the observed action is performed by another subject and that the predictive coding (PC) programs could start to work, allowing the acquisition of the action understanding ability.

*Modulation of associative and supervised learning based on basal ganglia dopamine regulation.* We propose that the basal ganglia might convey the dopamine reward signal to modulate both the associative/cortical and the supervised/cerebellar learning processes underlying the acquisition of action understanding by the mirror neuron system. During cortical associative learning when the observation of an action is correlated with its execution, an excitatory link between sensory and motor representations of the same action is built (Press et al., 2011b). During this sensorimotor learning the association between *context, location, and physical characteristics* of what is present in the first experience could be *reinforced* to boost the strength of the excitatory link established by the correlation between executed and observed action. The basal ganglia might participate to this reinforcement process. The basal ganglia are, indeed, the neural implementation of the “law of effect” responsible for sensorimotor learning that is reinforced by rewards, with the reward signal provided by dopamine in a gradual process of habit formation (Yin and Knowlton, 2006). Phasic dopamine signals acting on targets in the basal ganglia, limbic system, and cerebral cortex are critically involved in the required reinforcement process (Montague et al., 2004). The *dopaminergic* learning signal is supplied by the ventral tegmental area (VTA), which is under a strong control of basal ganglia and the hippocampus. Basal ganglia contribute to the production and modulation of dopamine learning signals on the basis of appetitive stimuli (Schultz, 2002). Hippocampus detects new information that is not already stored in long-term memory and on this basis it releases a *novelty signal* which is conveyed to the VTA where it contributes, along with salience and goal information, to the firing of dopamine cells (Lisman and Grace, 2005). In the case of mirror neurons, the information which would cause the release of dopamine might be represented by the observation of actions related to appetitive stimuli or to novel action outcomes never seen before.

The dopamine reward signal modulated by the basal ganglia might regulate learning in the cortico-cerebellar circuits as well. This proposal is supported by the recent data providing the anatomical substrate for a bidirectional communication between basal ganglia and cerebellum, introduced in Section 2.1 (Fig. 1d, dashed arrows; Hoshi et al., 2005; Bostan et al., 2010, 2013). We have seen there that the subthalamic nucleus of the basal ganglia has a substantial disynaptic projection to the cerebellar cortex. This pathway provides a means through which basal ganglia influence cerebellar function (Bostan et al., 2010). Moreover, the dentate nucleus of the cerebellum has a disynaptic projection to the main input stage of basal ganglia processing, the striatum (Hoshi et al., 2005). Taken together these results provide the substrate for an important two-way communication between basal ganglia and cerebellum, which parallels their indirect communication through the cerebral cortex (Houk, 2011). From a learning perspective, interconnecting a reinforcement learning module (pivoting on basal ganglia), and the related reward system, with a supervised learning module (pivoting on cerebellum) might lead to new computational operations where the reward dopamine signal modulated by the basal ganglia might regulate the effectiveness of supervised learning, elevating learning rates in rewarding contexts. This claim is in agreement with recent literature suggesting that the connection between basal ganglia and cerebellum provides an anatomical substrate for reward-related signals regulated by basal ganglia to influence cerebellar learning (Bostan et al., 2010, 2013; Thoma et al., 2008). Further support comes from imaging studies indicating the most intense cerebellar activations during the acquisition phase of conditioned forelimb movements (Milak et al., 1997), and when sensorimotor tasks are being learned (Friston et al., 1992; Imamizu et al., 2000).

## 4 Conclusion

The main message that this article conveys is that the sub-cortical processes driven by the cerebellum and the basal ganglia might support cortical structures, and in particular the mirror circuit, in expressing and acquiring action understanding capacities. In particular, evidence about the involvement of the *cerebellum* (e.g., Miall, 2003; Strick et al., 2009; Ito, 2008; Houk, 2011) and the *basal ganglia* (e.g., Middleton and Strick, 1994, 2000; Houk et al., 1996; Redgrave et al., 1999b; Cisek and Kalaska, 2010) in cortical functions has been used to support the idea that, within the *complete mirror system, cortical* and *sub-cortical structures* might work in concert to address three key open issues. These open issues concern how the motor abilities of an organism might contribute to action understanding; how mirror neurons might manage *multiple levels* at which an observed *action* can be understood; and how the action understanding capacity might be acquired. A direct consequence of our proposal is that impairments of cerebellar- and/or basal ganglia-cortical loops might lead to an impaired action understanding capacity, especially related to these three critical points. For example, impairments of the basal ganglia-cortical loops for action selection might affect the integration across the multiple levels at which an action can be understood, as well as action selection processes postulated by predictive coding. Similarly, the acquisition of action understanding capacity by the mirror neuron system might be impaired in cerebellar patients. Importantly, the plausibility of predictions similar to those proposed here has been recently confirmed by empirical studies aiming at demonstrating the impairment of some aspects of action understanding in cerebellar patients (e.g., Cattaneo et al., 2012; Sokolov et al., 2012) as well as in Parkinsonian subjects showing damages in some functions of basal ganglia (Alegre et al., 2010; Marceglia et al., 2009; Castiello et al., 2009). However, further research will be necessary to confirm these claims.
